# Amino Acid Substitutions Improve the Immunogenicity of H7N7HA Protein and Protect Mice against Lethal H7N7 Viral Challenge

**DOI:** 10.1371/journal.pone.0128940

**Published:** 2015-06-01

**Authors:** Subaschandrabose Rajesh kumar, Mookkan Prabakaran, Kattur Venkatachalam Ashok raj, Fang He, Jimmy Kwang

**Affiliations:** 1 Animal Health Biotechnology, Temasek Lifesciences Laboratory, 1 Research Link, National University of Singapore, Singapore, Republic of Singapore; 2 Department of Microbiology, Faculty of Medicine, National University of Singapore, Singapore, Republic of Singapore; US Food and Drug Administration, UNITED STATES

## Abstract

Avian influenza A H7N7/NL/219/03 virus creates a serious pandemic threat to human health because it can transmit directly from domestic poultry to humans and from human to human. Our previous vaccine study reported that mice when immunized intranasally (i.n) with live Bac-HA were protected from lethal H7N7/NL/219/03 challenge, whereas incomplete protection was obtained when administered subcutaneously (s.c) due to the fact that H7N7 is a poor inducer of neutralizing antibodies. Interestingly, our recent vaccine studies reported that mice when vaccinated subcutaneously with Bac-HA (H7N9) was protected against both H7N9 (A/Sh2/2013) and H7N7 virus challenge. HA1 region of both H7N7 and H7N9 viruses are differ at 15 amino acid positions. Among those, we selected three amino acid positions (T143, T198 and I211) in HA1 region of H7N7. These amino acids are located within or near the receptor binding site. Following the selection, we substituted the amino acid at these three positions with amino acids found on H7N9HA wild-type. In this study, we evaluate the impact of amino acid substitutions in the H7N7 HA-protein on the immunogenicity. We generated six mutant constructs from wild-type influenza H7N7HA cDNA by site directed mutagenesis, and individually expressed mutant HA protein on the surface of baculovirus (Bac-HA_m_) and compared their protective efficacy of the vaccines with Bac-H7N7HA wild-type (Bac-HA) by lethal H7N7 viral challenge in a mouse model. We found that mice immunized subcutaneously with Bac-HA_m_ constructs T143A or T198A-I211V or I211V-T143A serum showed significantly higher hemagglutination inhibition and neutralization titer against H7N7 and H7N9 viruses when compared to Bac-HA vaccinated mice groups. We also observed low level of lung viral titer, negligible weight loss and complete protection against lethal H7N7 viral challenge. Our results indicated that amino acid substitution at position 143 or 211 improve immunogenicity of H7N7HA vaccine against H7N7/NL/219/03 virus.

## Introduction

Prior to 2003, H7 subtype avian influenza virus causing human infection cases were very rare and mostly caused by occupational accidents or laboratory exposures [1–3]. For the last decade, more than 500 cases of human infections with H7 subtypes have been documented. Some of which include the H7N7 (NL/219/03) virus outbreak (89 human infected, one died) in the Netherlands and H7N9 outbreak (more than 440 human infected, out of which with 155 fatal cases) in China [4, 5]. H7N7/NL/219/03 virus is a highly pathogenic avian influenza that can infect both mice and ferrets without prior adaptation. Additionally, the H7N7/NL/219/03 viral attachment pattern and replication efficacy in mammalian respiratory tracts showed great similarity to H5N1 viruses [6]. Moreover, the replication patterns resembled that of H5N1 virus. The broad host spectrum, unusually high zoonotic potential, as well as its ability to suppress host immune responses in a similar way to 1918 H1N1 [7] virus are raising concerns for potential future influenza pandemics. Hence, the vaccine development against H7N7/NL/219/03 is of high priority to our defense against any possible H7 pandemic.

In our previous vaccine study, mice that were intranasally immunized with live Bac-HA were protected from lethal H7N7 viral challenge. However, no protection was observed when Bac-HA or inactivated H7N7 virus were administered subcutaneously, possibly due to diminished immunogenic nature of the H7N7 (H7N7/NL/219/03) virus [8–11]. The effect of glycan shielding on H7N7HA protein could be another plausible explanation too. Previous studies have reported that glycosylation of HAs could result in poor neutralizing antibody titer [12–14]. Both influenza and human immunodeficiency viruses (HIV) were found to employ glycan masking as a strategy for blocking antibody-epitope interactions [15–17]. Several studies have also explained the impact of HA glycosylation on the antigenicity, pathogenicity and evolution of influenza virus [18–22].

Interestingly, in our recent vaccine study, mice that were subcutaneously immunized with live Bac-HA (H7N9) survived in both H7N9 and H7N7 virus challenge [23]. Comparing H7N7HA1 and H7N9HA1 amino acid sequences, there were 15 amino acid positions differ were identified. Among these 15 positions, in this study we selected three positions, namely (i) 143, (ii) 198 and (iii) 211,(numbering of amino acid on HA sequence starts from ‘‘ATG” and includes the signal peptide) of the H7N7HA1 protein. These three positions are located within or near the receptor binding site of H7N7HA protein. Moreover, amino acid threonine at position 143 of NL/H7N7HA generated a potential N-linked glycosylation at position 141 of the H7N7 HA protein [24]. Following the selection, we generated a total of six mutant constructs by amino acid substitution at these three positions either singly (T143A, T198A and I211V) or doubly (T143A-198A, T198A-I211V and I211V-T143A) to the corresponding amino acids found in H7N9HA protein by site directed mutagenesis. The H7N7HA wild-type and all the H7HA mutants were expressed on the surface of baculovirus via Baculovirus Expression Vector System (BEVS). These HAs were further immunized subcutaneously into BALB/c mice, before they were intranasally challenged with mouse adapted HP RG-H7N7 virus. The immune responses and protections in BALB/c mice were then subsequently monitored to evaluate the efficacy of these expressed HAs as vaccines.

## Materials and Methods

### Ethics statement

All animal experimental protocols were reviewed and approved by the Institutional Animal Care and Use Committee (IACUC) of the Temasek Life Sciences Laboratory, National University of Singapore, Singapore. (IACUC approval number TLL-14-020). All animal experiments were carried out in animal BSL3 containment facility in compliance with CDC/NIH and WHO recommendations. Mice were housed in individually ventilated cages (Tecniplast Sealsafe) provisioned with water and standard food, and monitored daily for health condition. More than 25% body weight loss was used as criterion for early euthanasia. The animals were euthanized by CO_2_ inhalation for five minutes.

### Viruses and cells

Reassortant influenza virus RG-H7N7 (A/Netherland/219/03) and RG-H7N9 (A/Shanghai/2/2013) were generated by reverse genetics as described previously [25]. Viruses were propagated in 10 day old specific pathogen free embroyonated chicken eggs at 37°C. The tissue culture infectious dose 50 (TCID_50_) of reassortant viruses were calculated by the Muench-Reed method (1938) [26]. All experiments were performed in a biosafety level 3 (BSL-3) containment laboratory in compliance with CDC/NIH and WHO recommendations [27, 28].

Spodoptera frugiperda (Sf9II) cells (ATCC) were maintained in Sf900II serum free medium (Gibco BRL, USA) at 28°C for wild-type baculovirus (wt-Bac) and recombinant baculovirus production.

### Preparation of recombinant baculovirus subunit vaccine

The full length of H7N7HA (A/NL/219/03) cDNA gene was commercially synthesized based on the sequences from the NCBI influenza database (GenScript, USA). cDNA was used for Bac-HA and Bac-HA_m_ constructs preparation. For preparing the mutant constructs (single mutants T143A, T198A, I211V, double mutants T143A-T198A, T198A-I211V and I211V-T143A), we substituted the amino acid alanine at 143, alanine at 198 and valine at 211 coding gene inH7N7HA wild-type cDNA by using quick change site directed mutagenesis kit (Agilent Technologies). Transfection and viral amplification were carried out according to the Baculovirus Expression System Manual (Invitrogen Life technologies).The live baculovirus content of each vaccine constructs were determined by TCID50 method as described Reed and Munch (1938)[26]. HA content of the each vaccine construct was determined densitometrically by comparing against known concentrations of the insect cells expressed H7N7HA protein(Sino Biologic, Beijing, China) using ImageJ (National Institutes of Health) as described Miriam et al.(2014)[29].

### Immunofluorescence assays to detect HA expression in insect cells

The Sf9 cells were infected with Bac-HA or Bac-HA_m_ constructs or wt-Bac at 0.5 MOI. Two days post infection, the cells were fixed with 4% formaldehyde and stained with mouse anti H7N7-HA monoclonal antibody (8H9), followed by FITC conjugated goat anti mouse immunoglobulin (Dako Cytomation, Denmark). The fluorescence signal was detected with an inverted fluorescence microscope (Olympus, UK) and the images were captured by a digital imaging system (Nikon, USA).

### Glycosylation pattern confirmation by western blot analysis

Amino acid substitution at T143A in H7N7HA wild-type protein inhibits synthesis of N- glycosylation motif in A141. It was confirmed by western blot analysis. Briefly, the Bac-HA or Bac-HA mutant infected cell supernatant was mixed with Laemmli sample buffer and resolved in 12% SDS–PAGE. The gel was transferred to a nitrocellulose membrane and Western blotting was performed as described previously Kumar et al (2013)[8].The anti-mouse rHA polyclonal antibody (TLL, Singapore) at a dilution of 1:250 was used as the primary antibody and rabbit anti-mouse Ig (Dako Cytomation, Denmark) at a dilution of 1:3000 was used as a secondary antibody. The protein bands were visualized by chemiluminescence kit (Amersham, UK).

### Mice immunization

Six to eight weeks old specific pathogen free female BALB/c mice were used in all experiments. Mice (10 mice per group/9 groups) were subcutaneously immunized with 100 μL of purified live Bac-HA or Bac-HA_m_ constructs (T143A or T198A or I211V, T143A-T198A or T198A-I211V or I211V-T143A) containing 2.5μg of HA content (approximately1 X10^7^to 1X10^7.5^ pfu/ml recombinant Baculovirus—Live baculovirus content of each recombinant constructs were determined by using TCID50 method as described Reed and Munch (1938) [26]) or wt-Bac (10^8^ PFU) and PBS control. Four weeks after the first immunization, a second dose of vaccine was given to all immunized mice groups. Two weeks after the second immunization (42^nd^day), serum samples were collected from each experimental mice group (Five mice/Group). All samples were stored at -20°C for measuring the hemagglutination inhibition (HAI) and microneutralization (MN) assay against RG-H7N7, RG-H7N9 viruses.

### Microneutralization (MN) assay

The MN assay was performed as described previously [30]. Briefly, serial two fold dilutions of heat inactivated (56°C for 45 min) 42nd day immune serum samples were mixed separately with 100 TCID_50_ of RG-H7N7 or RG-H7N9 viruses and incubated at room temperature for 1 h. The mixtures were added to the MDCK monolayers in triplicate wells. One hour after infection, the virus-serum mixture was removed, and serum free DMEM was added to each well. Cytopathic effect was observed 3 to 4 days after incubation at 37°C. The highest serum dilutions that completely protected cells from cytopathology were considered to be the MN titer.

### Hemagglutination inhibition (HAI) assay

Functional serum antibody titers were determined by hemagglutination inhibition assay [31].Serum samples were treated with 4 volumes of a receptor-destroying enzyme of *Vibrio cholera* filtrate (RDE Denka Siken Co., Japan) for 18 h at 37°C. After addition of 3 volumes of 2.5% (v/v) sodium citrate, the serum samples were incubated at 56°C for 30 min and diluted with PBS to yield a 1:10 dilution of the original serum sample. Serum samples were 2-fold serially diluted in PBS (25 μL sample volume) in Nunc 96-well polystyrene V-bottom microwell plates (Thermo Fisher Scientific, Denmark). Approximately four HA units of each H7 subtype (RG-H7N7 or RG-H7N9) viral antigen was incubated with the serum for 30 min at room temperature followed by the addition of 1% chicken RBCs and incubation at room temperature for 40 min. The inhibition of hemagglutination at the highest serum dilution was considered the HAI titer of the serum.

### Mice viral challenge

To assess the protective efficacy of the vaccines, immunized mice groups were anesthetized intraperitoneally with ketamine (100 mg/kg)/Xylazine (20 mg/kg) and intranasally challenged with 50 μL (25 μL per naris) of 5 MLD_50_ of mouse adapted RG-H7N7 virus. Mice were observed daily to monitor body weight, clinical signs of disease (ruffled fur, lack of mobility, labored breathing) and mortality. Mice were humanely euthanized with CO_2_ inhalation if their body weight dropped to 75% of baseline weights. For determination of lung viral titers after challenge, three mice from each vaccinated group was euthanized by CO_2_ inhalation on day 5 post-challenge as described Prabakaran et al. [23].

### B cell and T cell epitope prediction

The immune epitope Database (IEDB) analysis resources were used [32] for predicting the B—cell and T-cell epitopes in hemagglutinin molecule of A/Netherland/219/2003 and H7N9 A/Shanghai/2/2013 viruses (Table 1) for searching the cross reactive epitopes between the viruses.

**Table 1:** Predicted T cell and B cell epitopes in HA molecules of H7N7, H7N9 influenza viruses.

Start	End	Sequence		Epitope
		(H7N9)	(H7N7)	
138	150	IRTNGATSACRRS	IRTNGTTSACRRS	B cell
194	205	SVSTAEQTKLYG	SGSTTEQTKLYG	B cell
203	211	LYGSGNKLV	LYGSGNKLI	T cell

The differences between HA amino acid sequence of H7N9 and H7N7 influenza have been underlined.

### Statistical analysis

All data are expressed as arithmetic mean ± standard deviation. The significance between the groups was calculated using unpaired two-tailed Student’s t test and significance was expressed as ** *P*<0.001.

## Results

### Baculovirus-HA vaccine construction

**Fig 1:**
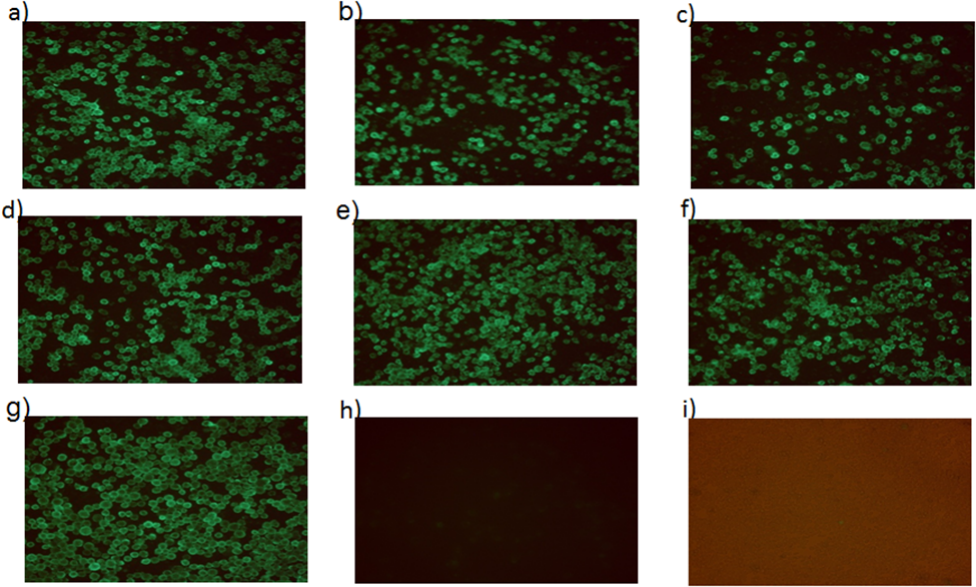
Confirmation of baculovirus displayed H7N7HA and H7N7HA mutant in insects cells by immunofluorescence assay. The Sf9 cells were infected with Bac-HA_m_ constructs; a) T143A or b) T198A, or c) I211V or d) T143A-T198A or e) T198A-I211V or f) I211V-T143A or g) Bac-HA or h) wild-type baculovirus (wt-Bac), or i) Control Sf9 cells. 48 h post infection the cells were fixed and analyzed by mAb specific to H7N7 influenza virus.

The Bac-HA and other Bac-HA_m_ protein expressions were analyzed by immunofluorescence staining and their native structure were analyzed by hemagglutination activity with Chicken RBCs. Fluorescence signal and agglutination activity (32 HA units) were detected only in baculovirus expressed H7N7HA (Bac-HA or Bac-HA_m_ constructs) infected cells. In contrast, no fluorescence signal and agglutination activity was observed in cells infected with wt-Bac (Fig 1).

### Western blot confirmation of HA protein with lacking of N-linked glycosylation

**Fig 2:**
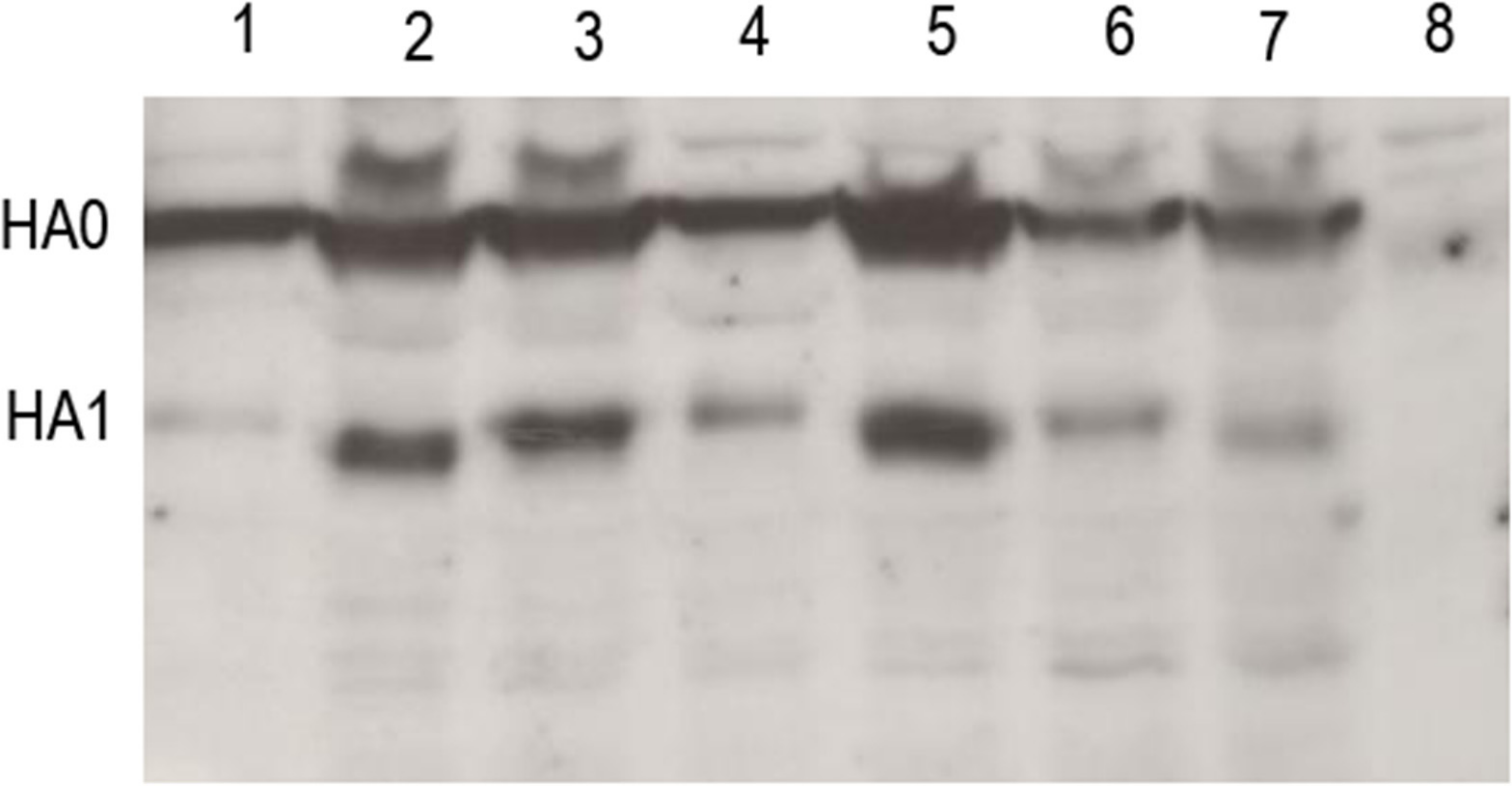
Western blot analysis of baculovirus displayed H7N7HA and H7N7HA mutant protein. All the samples were reduced with loading dye containing β-mercaptoethanol reducing agent. Lane 1: H7N7HA wild-type; Lane 2:T143A; Lane 3:T198A; Lane 4:I211V; Lane 5:T143A-T198A; Lane 6:T198A-I211V; Lane 7: I211V-T143A; Lane 8, wt-Bac.

Amino acid substitution at position 143T inhibits synthesis of N linked glycosylation motif in insect cell expressed H7NHA protein which was confirmed by western blot through molecular weight differentiation (Fig 2). The result showed that molecular weight of single mutated (T143A) or double mutated (T143A-T198A, T143A-I211V) HA protein was reduced (approximately 64–66 kDa) compared to wild-type HA protein (approximately 67–70kDa). However, other mutated HA protein (T198A or I211V or T198A-I211V) did not show any differences in molecular weight. This shows that amino acid substitution at T143A result in lack of N-glycosylation motif in A141.

### B cell and T cell epitopes prediction

We predicted B-cell and T-cell epitope in HA molecule of H7N7 and H7N9 viruses by using immune epitope analysis resources as described previously [32, 33]. Here, we showed B-cell and T-cell epitope located in the mutant region (143,198 and 211) of HA molecule (Table 1). Based on the epitope prediction result, both viruses have a B-cell epitope at region 138–150 (T143A), 194-205(T198A) and T-cell epitope at position 203–211.

### Systemic immune response

**Fig 3:**
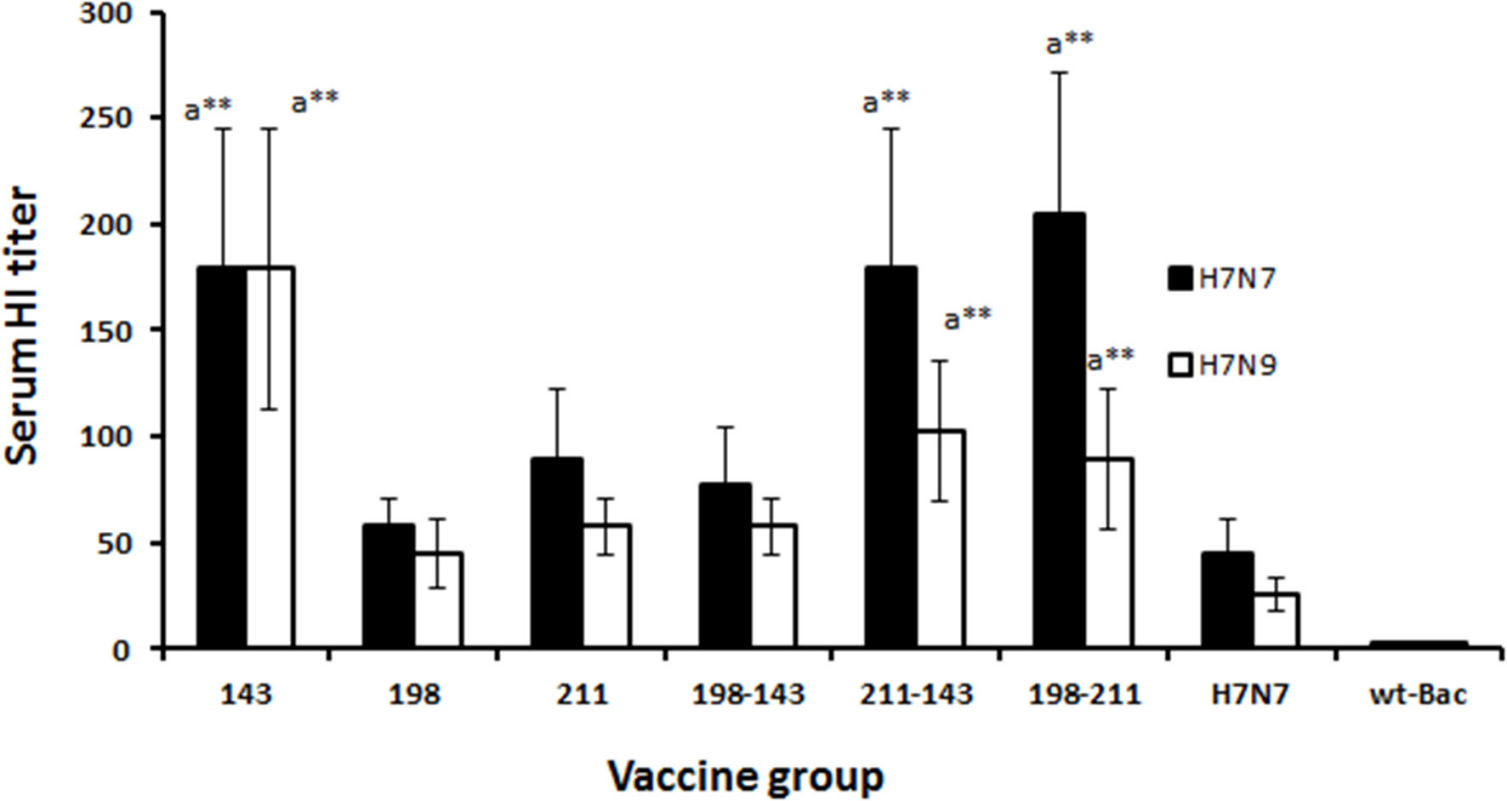
Serum hemagglutination inhibition (HAI) activity of sera obtained from subcutaneous immunizations on day 42. Groups of mice (n = 10) were subcutaneously immunized with a constant dosage of 100 μl containing 2.5 μg of live Bac-HA, or Bac-HA_m_ constructs T143A or T198A or I211V or T143A-T198A or T198A-I211V or I211V-T143A or wild-type baculovirus (PFU 1X10^8^), controls on day 0 and 28. Serum samples were collected on day 42 from each experimental mice group (Five mice/Group) for measuring the serum hemagglutination inhibition (HI) titer. Four HA units of RG-H7N7 (A/Netherlands/219/03), RG-H7N9 (A/Shanghai/2/2013), virus were used for HA inhibitory study. ^a^-when compared with wild-type H7N7 Bac-HA vaccine group. Each point represents the arithmetic mean value (n = 5) ± SD (**P<0.001).

The functionality of serum antibodies was tested in hemagglutination inhibition (HAI) assay (Fig 3). Sera of mice immunized s.c. with Bac-HA_m_ construct I211V-T143A exhibited slightly enhanced HAI titer but no significant difference compared to mice immunized with T143A or T198A-I211V Bac-HA_m_ construct. However, significant difference was observed between mice immunized with Bac-HA_m_ construct T143A or I211V-T143A or T143A or T198A-I211V groups and Bac-HA immunized mice groups (Fig 3).

**Fig 4:**
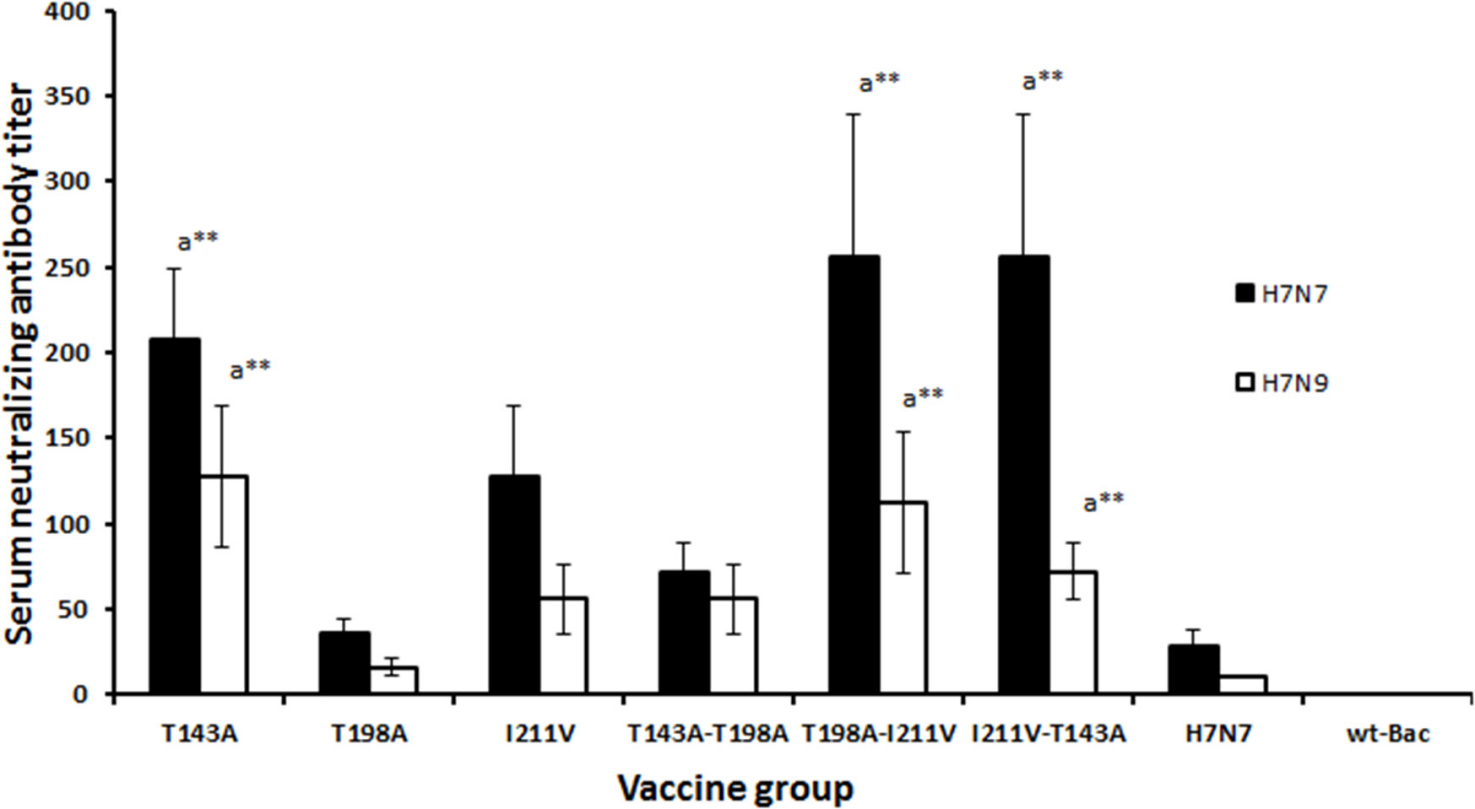
Microneutralization titers of vaccinated mouse sera against 100TCID_50_ of RG-H7N7 and RG-H7N9 viruses. Groups of mice (n = 10) were subcutaneously immunized with a constant dosage of 100 μl containing 2.5 μg of live Bac-HA, or Bac-HA_m_ constructs T143A or T198A or I211V or T143A-T198A or T198A-I211V or I211V-T143A or wild- type baculovirus (PFU 1X10^8^), controls on day 0 and 28. Serum samples were collected on day 42 from each experimental mice group (Five mice/Group) for measuring the serum neutralization antibody titers against H7N7 (A/Netherlands/219/03) or H7N9 (A/Shanghai/2/2013) viruses. Neutralizing titers are arithmetic mean of the highest dilution of serum which yielded a 50% reduction in virus infectivity. a-when compared with wild-type H7N7 Bac-HA vaccine group. Each point represents the arithmetic mean value (n = 5) ± SD (**P<0.001).

Further, the ability of the serum antibodies to neutralize live influenza virus was tested in a microneutralization assay (Fig 4). The neutralizing antibody titers of sera from s.c. immunized mice groups at day 42 after the first immunization were measured against 100 TCID_50_ of homologous H7N7 reassortant virus. The sera obtained from the s.c. vaccinated Bac-HA_m_ construct T143A (1:208), T198A-I211V and I211V-T143A showed a higher neutralizing titer (1:256) against RG-H7N7virus compared to other vaccinated groups. Also, we tested cross-neutralization activity of vaccinated sera against RG-H7N9 virus (Fig 4). Interestingly Bac-HA_m_ construct T143A(1:128) or T198A-I211V (1:112) vaccinated group showed a higher neutralization activity against H7N9 compared to other vaccinated groups but no significant difference compared to mice immunized with T143A-I211V. Bac-HA vaccinated mice group showed significantly low level of neutralization activity to H7N7 (1:28) or H7N9 (1:10) RG viruses compared toT143A or T198A-I211V or T143A-I211V Bac-HA_m_ construct vaccinated group (Fig 4).

### Mice challenge study

**Fig 5:**
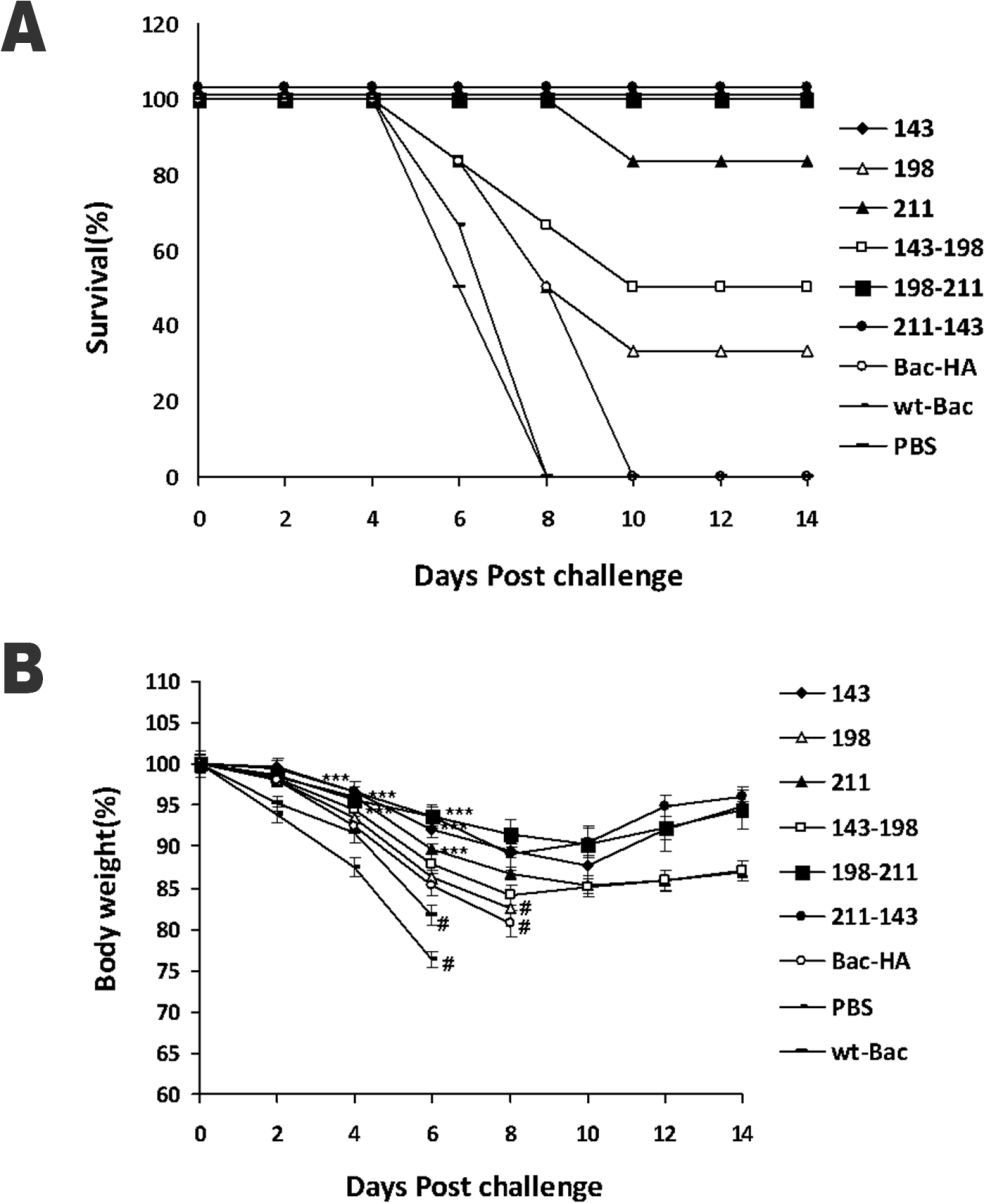
Protection of mice from lethal H7N7 influenza virus challenge. Mice (n = 10/groups) were immunized subcutaneously on day 0 and 28. All groups were challenged intranasally with 5 MLD_50_ of mouse-adapted H7N7 virus on day 49. Mice were monitored for survival for 14 days and the results were expressed in percent survival (5A). Weight loss of the mice groups was also monitored throughout the 14 day observation period and the results were expressed in percent body weight compared to the beginning of the trial (5B). Each point represents the arithmetic mean (n = 6) value ± SD (***P<0.0001, when compared to Bac-HA vaccinated mice group); # represents ≥33.3 survival in the group.

**Fig 6:**
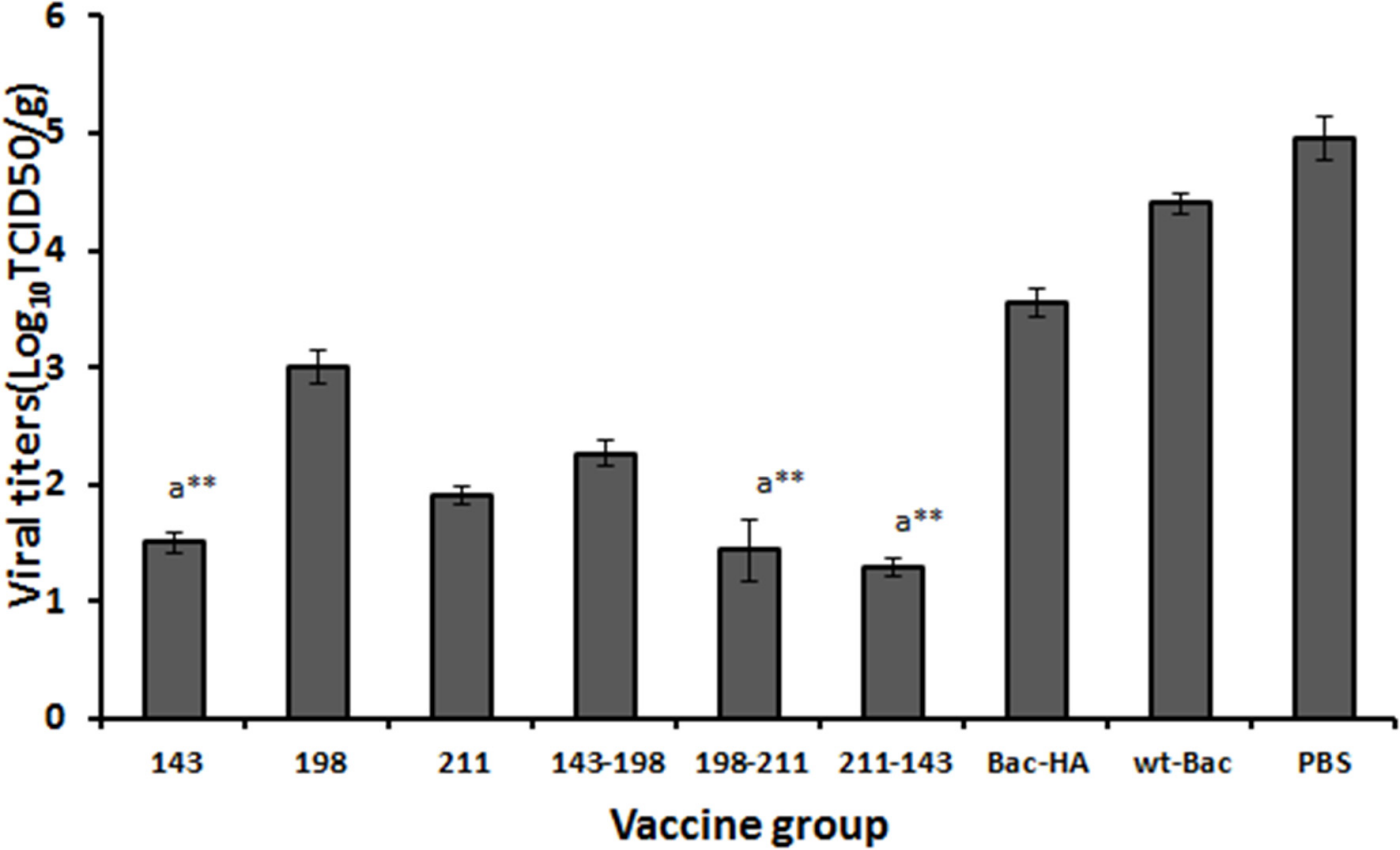
H7N7 influenza viral titers in lungs at 5 day post challenge following subcutaneous immunization. Viral titer measurement in lungs of at 5 day post challenge following mice challenged with 5MLD_50_ of H7N7 influenza virus after subcutaneous immunization. The results of viral titers in lungs were expressed in terms of mean value of log_10_TCID_50_/g (n = 3) ± standard deviation (SD). (^a^-when compared with wild-type H7N7 Bac-HA vaccine group, ** *P*<0.001).

The mice viral challenge study result showed that s.c. immunized with Bac-HA_m_ construct T143A or T198A-I211V or I211V-T143A mice groups achieved 100% protection throughout the 14 day observation period with a negligible weight loss (Fig 5A, Fig 5B). Other Bac-HA_m_ constructs T198A (33.33%), I211V (83.33%), and T143A-T198A (50%) also give partial protection against lethal H7N7 challenging, but Bac-HA administered by s.c. route, failed to protect the mice (Fig 5A). These animals showed obvious clinical symptoms such as ruffled fur and hunching shoulders which started at day 4 post challenges. Also, a significant weight loss of up to 25% to 35% was observed before they were sacrificed at day 10 (Fig 5B). Also, the mice lung viral titer result showed that the H7N7 influenza virus titer was significantly reduced in the s.c. immunization with Bac-HA_m_ construct T143A or I211V or T198A-I211V or I211V-T143A groups compared to other Bac-HA_m_ vaccinated or Bac-HA vaccinated groups (Fig 6).

## Discussion

Avian influenza viruses, particularly H7 subtype, have been responsible for large outbreaks of diseases among poultry, as well as in human beings, in recent years [1, 2]. In our previous vaccine study, mice subcutaneously immunized with live baculovirus or inactivated RG-H7N7 virus did not provide protection against the lethal H7N7 influenza virus. Such lack of protection could be due to the less immunogenic nature of H7N7 virus [8–11]. Additionally, several previous studies have suggested that amino acid substitutions in critical region of the antigen improve immunogenicity [34, 35]. In our studies, the amino acid position 143, 198 and 211 of H7N7HA protein were selected and substituted, either singly or doubly, to the corresponding amino acids found on H7N9HA wild-type sequence using site directed mutagenesis. Six H7N7 HA mutant constructs, namely the single mutants: (i) T143A, (ii) T198A and (iii) I211V, and the double mutants (iv) T143A-T198A, (v) T198A-I211V, (vi) I211V-T143A) were generated. All these constructs were expressed on the baculovirus surface using insect cell line and the protective efficacy of these vaccine constructs were evaluated using a mouse model, where the mice were challenged with RG-H7N7 viruses.

The HAI activity of the sera derived from mice vaccinated against H7N7 virus were subsequently tested. The sera of the mice vaccinated with Bac-HA_m_ constructs T143A, I211V, T198A-I211V and T143-I211V showed higher HAI titers when compared to that of mice vaccinated with other Bac-HA_m_ constructs. These higher HAI titer observed could be the result of the respective mutations being located within or near the receptor binding site, or even in antigenic dominant region of the HA. In support of our explanation, Medina et al. [18] has previously reported that adding one or two glycosylation on residues around dominant antigenic region was sufficient to significantly reduce the HAI activity of the polyclonal response elicited by the wild-type unglycosylated virus.

This higher HAI titer was also true when we tested the neutralizing activity of sera obtained from mice immunized with Bac-HA_m_ constructs. The result showed Bac-HA_m_ constructs (T143A, T198-211, and I211V-T143A) had significantly higher neutralizing activity against homologous RG-H7N7 virus when compared to the sera from mice vaccinated with Bac-HA and other Bac-HA_m_. This higher neutralizing response against H7N7 virus could be attributed to the amino acid substitution at position 143, which inhibits the N-linked glycosylation with Asn141 in mutant HA (143A) protein. The lack of glycosylated motif may, in turn, expose the hidden neutralizing epitope at region 138–150 (Table 1), thus, inducing a higher neutralizing antibody titer. On the contrary, wild-type HA protein has the potential to glycosylate at position 141, creating a motif region 138–150 (Table 1), thereby hiding the neutralization epitope. This, therefore, leads to a low neutralizing antibody titer. Supporting our hypothesis, Ma et al. [36] has reported that partially deglycoslated envelope would bind better than fully glycosylated gp41 envelope to specific naıve B cells, hence improving immunogenicity. Interestingly, the sera of the Bac-HA_m_ construct I211V vaccinated mice also showed higher HAI and neutralization titer against H7N7 virus than that of Bac-HA. The exact mechanism as to how H7N7HA mutant (211V) induces higher HAI and neutralization titer is yet to be known and we hope that the mechanism could be elucidated in our future studies.

A significant association between complete protection against RG-H7N7 influenza virus and titers of HI and neutralization antibody was observed. Previous study has also shown that higher neutralizing antibody and HI titer does protect mice from H7N9 [37], hence, supporting the association seen in our experiments. Cross neutralizing activity was analyzed by MN assay against virus of H7 subtype H7N9. Sera of mice immunized with Bac-HA_m_ constructs T143A, T198A-I211V and T143A-T211A showed notably higher neutralizing activity against RG-H7N9 virus than those sera drawn from mice vaccinated using Bac-HA and other Bac-HA_m_. Based on the epitope prediction result (Table 1, courtesy: Larisa Rudenko et al 2013) [33], both H7N7and H7N9 viruses have a B-cell epitope at region 138–150 and T-cell epitope at position 203–211. The difference between the amino acid sequence of the B-cell epitope of H7N9 and H7N7 is at position 143. Amino acid Thr at position 143 of NL/H7N7HA generated an N-linked glycosylation with Asn141 [38], creating a glycan mask on the predicted B-cell epitope located at position between 138 and 150. H7N9 virus, on the other hand, have alanine at position 143, and this inhibits the N-linked glycosylation. The unglycosylated motif of H7N7HA_m_ (T143A) protein, similar to those found in H7N9 virus, results in the exposure of the B-cell epitope, between region 138 and 150. This explains why antiserum of mice vaccinated with H7N7_m_ constructs has higher cross neutralizing activity against H7N9 virus.

Based on T-cell epitope prediction, H7N7 and H7N9 viruses, have a T-cell epitope at position 203–211 of their HA proteins. One difference between the amino acid sequence of H7N9 and H7N7 T-cell epitope is at position 211. Several previous studies have suggested that T-cell epitope containing amino acid valine at position 9 acts as a dominant C-terminal anchor residue that provides higher binding affinity to MHC, hence inducing greater antigen-specific T cell immune responses [39–42]. Similarly, H7N7HA mutant I211V carrying the amino acid valine (at position 9 in its T cell epitope) may provide such greater MHC binding affinity, thereby, inducing higher peptide-specific T-cell immune responses. This might be the reason for the cross-protection against H7N9 influenza virus when immunized with I211V of H7N7HA.

The protective efficacy of vaccine candidates against RG-H7N7 influenza virus was determined in a mouse challenge study. In this study, subcutaneous vaccinations using Bac-HA_m_ constructs T143A, I211V-T143V and T198A-I211V completely protects the mice that were challenged with 5MLD50 of RG-H7N7 influenza virus. The complete protection observed could be the effect of higher HAI titer and neutralization antibody titer against H7N7 influenza virus. In addition to the 3 constructs just mentioned, vaccinations using 2 other Bac-HA_m_ constructs T198A and T143A-T198A have provided 33.3% and 50% protection respectively. Moreover, immunization of mice using mutant (I211V) HA construct provides sufficient protection (83.33%) when challenged with RG-H7N7. Such reasonable protection could be the effects of higher T-cell responses against H7N7HA. In contrast, no protection was observed in mice vaccinated with Bac-HA, likely a result of the significantly lower level of HAI titer induced by subcutaneous vaccination with Bac-HA construct. Furthermore, the neutralizing titer of sera, from mice vaccinated with Bac-HA_m_ constructs T143A, T198A-I211V and T143A-I211V were significantly higher than that of Bac-HA vaccinated mice (Fig 4). This, therefore, shows a significant correlation between neutralization titer and animal protection. Besides the higher neutralizing titer, mice vaccinated subcutaneously with Bac-HA_m_ constructs T143A, T143A-I211V, and T198A-I211V also showed a reduced lung viral titer, compared to all other immunized groups (Fig 6). This reduced titer could be the effects of successful induction of neutralizing antibodies. In summary, mice that were subcutaneously vaccinated with Bac-HA_m_ constructs T143A, T143A-I211V and T198A-I211V induced higher HAI and neutralizing antibody titers than those that were Bac-HA vaccinated. Such higher HAI and cross-neutralizing antibody titers, in turn, provide complete protection against RG-H7N7influenza virus.

In conclusion our results indicated that amino acid substitution at positions T143A or I211V significantly enhance the immunogenicity of H7N7HA vaccine against H7N7 and protect mice from lethal H7N7/NL/219/03 virus challenge. Also, we needed further studies to know the role of other amino acid positions and substitution of other amino acids at the same position on immunogenicity of H7HA antigen.

Our findings suggest that we can enhance the immunogenicity of poorly immunogenic conventional influenza vaccines (live attenuated or heat killed) or subunit vaccines or viral like particle vaccines by identifying and inhibiting the potential glycan motif-epitope masks formation in HA protein by amino acids substitution. Even though we could not provide more and precise mechanism regarding how I211V mutation enhancing the immunogenicity, the animal protection against lethal challenge with H7N7 virus data suggest that we can improve antigen specific T cell efficacy by amino acids substitution at T cell epitopes, which provide higher binding affinity to MHC and induce higher antigen specific T cell response.
